# Biopsy bacterial signature can predict patient tissue malignancy

**DOI:** 10.1038/s41598-021-98089-3

**Published:** 2021-09-17

**Authors:** Glenn Hogan, Julia Eckenberger, Neegam Narayanen, Sidney P. Walker, Marcus J. Claesson, Mark Corrigan, Deirdre O’Hanlon, Mark Tangney

**Affiliations:** 1 Cancer Research@UCC, University College Cork, Cork, Ireland; 2 SynBioCentre, University College Cork, Cork, Ireland; 3 APC Microbiome Ireland, University College Cork, Cork, Ireland; 4 School of Microbiology, University College Cork, Cork, Ireland; 5 General Surgery, Cork University Hospital, Cork, Ireland; 6 General Surgery, South Infirmary Victoria University Hospital, Cork, Ireland

**Keywords:** Computational models, Genome informatics, Machine learning, Predictive medicine, Software, Breast cancer, Tumour biomarkers, Cancer, Computational biology and bioinformatics, Microbiology, Applied microbiology, Bacteria, Biomarkers, Diagnostic markers, Predictive markers

## Abstract

Considerable recent research has indicated the presence of bacteria in a variety of human tumours and matched normal tissue. Rather than focusing on further identification of bacteria within tumour samples, we reversed the hypothesis to query if establishing the bacterial profile of a tissue biopsy could reveal its histology / malignancy status. The aim of the present study was therefore to differentiate between malignant and non-malignant fresh breast biopsy specimens, collected specifically for this purpose, based on bacterial sequence data alone. Fresh tissue biopsies were obtained from breast cancer patients and subjected to 16S rRNA gene sequencing. Progressive microbiological and bioinformatic contamination control practices were imparted at all points of specimen handling and bioinformatic manipulation. Differences in breast tumour and matched normal tissues were probed using a variety of statistical and machine-learning-based strategies. Breast tumour and matched normal tissue microbiome profiles proved sufficiently different to indicate that a classification strategy using bacterial biomarkers could be effective. Leave-one-out cross-validation of the predictive model confirmed the ability to identify malignant breast tissue from its bacterial signature with 84.78% accuracy, with a corresponding area under the receiver operating characteristic curve of 0.888. This study provides proof-of-concept data, from fit-for-purpose study material, on the potential to use the bacterial signature of tissue biopsies to identify their malignancy status.

## Introduction

High-throughput 16S rRNA gene sequencing has recently been used to describe the microbial communities of in vivo compartments that were up until that point described as “sterile”. Among these is the microbiome of the human breast, for which fresh tumour and matched normal tissues have been characterised by our group^1–3^ and substantiated by other investigators^4–7^. Some of these studies have attempted to define significant differences between breast tumour and matched normal tissues in terms of their overall bacterial profiles, but have been largely unsuccessful ^4,5,7^. Nonetheless, these preliminary analyses have invited discussion and evaluation of the wider relevance of these data, such as their utility within medical and diagnostic contexts.

Recently, a comprehensive microbiome analysis across 33 cancer types suggested that differences in bacterial diversity exist between malignant and healthy tissues, as well as between different cancer types^8^. This presents the possibility of exploiting such differences diagnostically. However, these findings are based on microbiome data drawn from tissues that were collected for The Cancer Genome Atlas (TCGA) project. These specimens are potentially unsuitable for analysis of microbial DNA, due to a high likelihood of contamination, a lack of negative controls, and DNA extraction techniques that are incompatible with bacterial cells^9^. While robust bioinformatic contamination control was applied to these microbiome data, no method exists that can decontaminate samples completely, in silico. Furthermore, efforts to characterise the breast microbiota suggest that breast tissues are low-biomass specimens^3,10^, which are especially prone to undue influence by contamination^11^.

The apparent low biomass of breast tissue has created ambiguity in breast microbiome studies, given the limitations that deep sequencing techniques have in relation to low-biomass samples. The revelation that DNA extraction kits contain bacterial DNA^12^ reinforces concern that low-biomass samples may be especially affected by kit contaminants if samples are not handled properly. Furthermore, a review of the sample collection protocols of breast microbiome studies reveals that inconsistencies arise here. Some studies utilise negative controls that aim to capture environmental contamination of samples that might arise in the operating theatre, while omitting controls that would indicate contamination originating from the patient's skin^3–5^, while other studies control for the reverse^6^, and one study utilised both skin and environmental contamination controls^2^. Additionally, investigators of the breast microbiome often report relatively high levels of sample manipulation prior to DNA extraction, including excision of the breast specimen, followed by further handling in a pathology laboratory^4,6^.

Although its mortality is decreasing, breast cancer remains the second most common cause of cancer death in women after lung cancer, and invasive breast cancer will afflict 1 in 8 women over a lifetime^13^. Breast health is therefore still a key concern, and this is reflected in the myriad publications that aim to mobilise efforts to improve screening and diagnoses of breast cancer and, indeed, define its microbiome. However, the above factors have each stifled research in this field, as some studies analysing breast tissue, and low-biomass material in general, have been criticised for taking insufficient precautions in limiting the effect that environmental contamination may have on the data^11^. Acknowledging the proneness to contamination that breast specimens may have, minimising human interaction with them prior to analyses, and adopting appropriate analytical measures, is apt. Thus, the approaches described below aim to approach with greater sensitivity the potential sources of contamination that can come to bear at many points of specimen collection and processing.

Despite the above complications, data on the tumour microbiome to date indicate the potential for a new class of bacteria-based oncological biomarkers. To expand on this, we wished to examine if microbiome-based detection of malignancy is still effective when the confounding factors listed above have been accounted for, in an ‘in-practice’ setting (biopsies). The aim of the present study, therefore, was to derive high-quality bacterial profile data from fresh biopsy specimens, collected specifically for this purpose, to examine bacterial signature as a predictor of patient tissue malignancy.

## Results

### Bespoke tissue collection strategy produces high-quality sequence data

**Figure 1:**
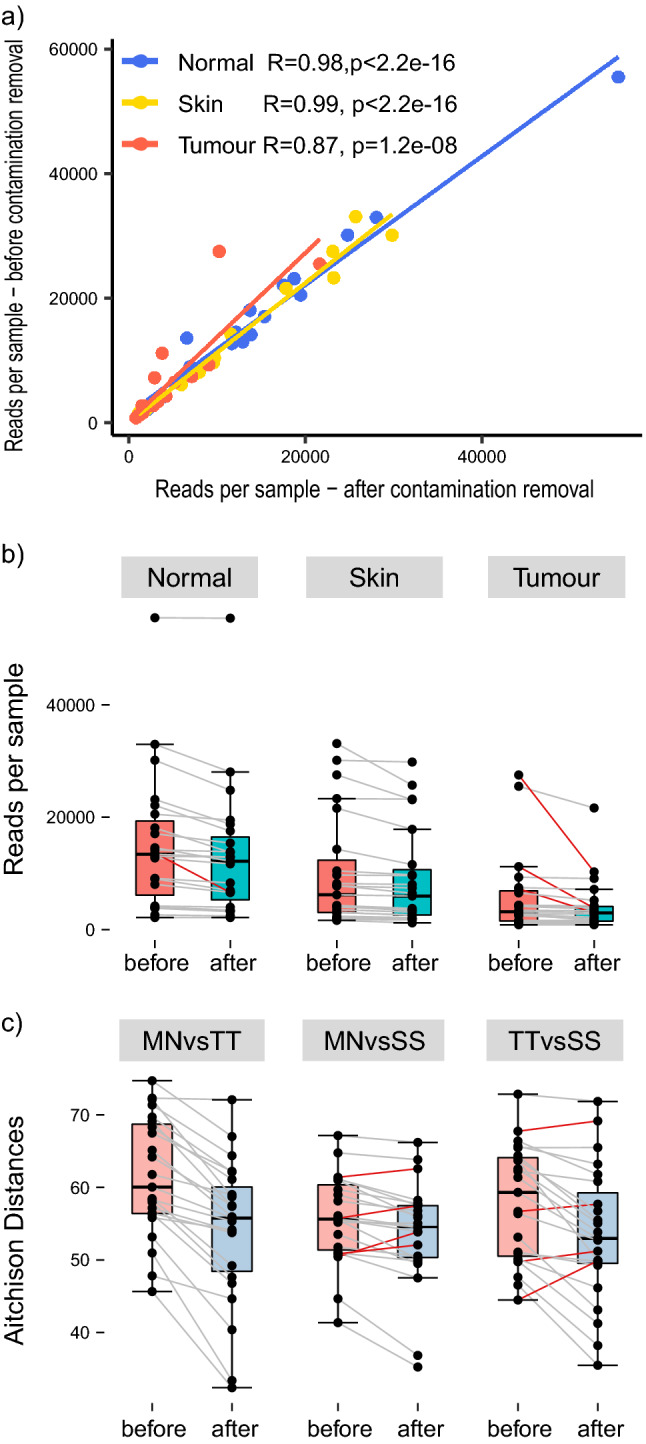
Investigation of the effect of contamination removal on the number of sequencing reads per patient sample. **(a)** Correlation of reads per sample by tissue type prior to, and following, contamination removal. (**b)** Box plots of reads per sample by tissue type, prior to, and following, contamination removal. Red lines indicate samples that lost more than half their total reads following contamination removal. (**c)** Calculation of pairwise distances, before and after contamination removal, between tumour tissue (TT), matched normal tissue (MN), skin swabs (SS).

As the biopsies under study are low-biomass specimens, it was necessary to remove human-genome-aligning reads^14^, and ensure that any biological signal was not distorted by environmental contaminants or by inter-patient variation. SourceTracker (v1.0)^15^ indicated low-to-moderate levels of contamination, which was subsequently removed with Decontam (v1.0.0)^16^ (Fig. 1), per published guidelines ^9^. For only four samples, more than half the sequencing data comprised contaminants (Fig. 1b). The strong correlation between numbers of sequencing reads before and after contamination removal reinforces the deduction, facilitated by the SourceTracker algorithm, that the biological signal of these samples has not been significantly distorted during collection and processing, increasing the likelihood of identifying genuinely distinct microbial signatures that are specific to malignant tissue.

Prior to contamination removal, 714,392 sequencing reads were available for analysis, equating to 10,353 ± 2352 reads per sample, on average. Following removal, 605,852 reads remained, equating to 8780 ± 2272 reads per sample, on average. Pairwise distances of samples taken from the same patient decreased after contamination removal in all but 9 samples. Hence, removing contamination can potentially improve the discriminability of samples between sampling sites (Fig. 1c).

### Differentially abundant bacteria exist between breast tumour and matched normal tissues, and skin surface swabs

**Figure 2:**
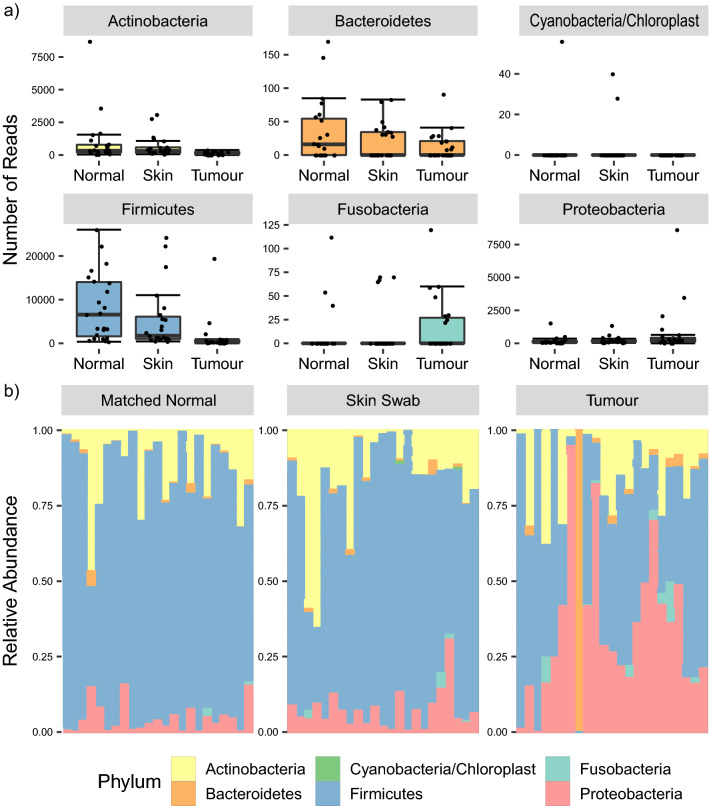
Composition of breast cancer patient specimens in terms of bacterial phyla. **(a)** Comparison of abundances of various bacterial phyla between patient sampling sites. (**b)** Sample composition at the phylum level, grouped by patient sample type.

Sample composition plots at phylum level indicated elevated numbers of Proteobacteria and Fusobacteria, and decreased numbers of Firmicutes, in tumour samples compared with matched normal tissue and skin swabs (Fig. 2). Limited differences between matched normal tissue and skin swabs were observed in terms of sample composition. The Dirichlet-Multinomial test comparison confirmed this, by failing to reject the null hypothesis of no significant difference between skin swabs versus matched normal tissue (Xdc:− 1.99, *P* = 1), while the comparison of tumour tissue with both skin swabs and matched normal tissue showed statistically significant differences (Xdc:33.82, *P* = 7.3e−6; Xdc:44.89, *P* = 4.9e−8, respectively).

**Figure 3:**
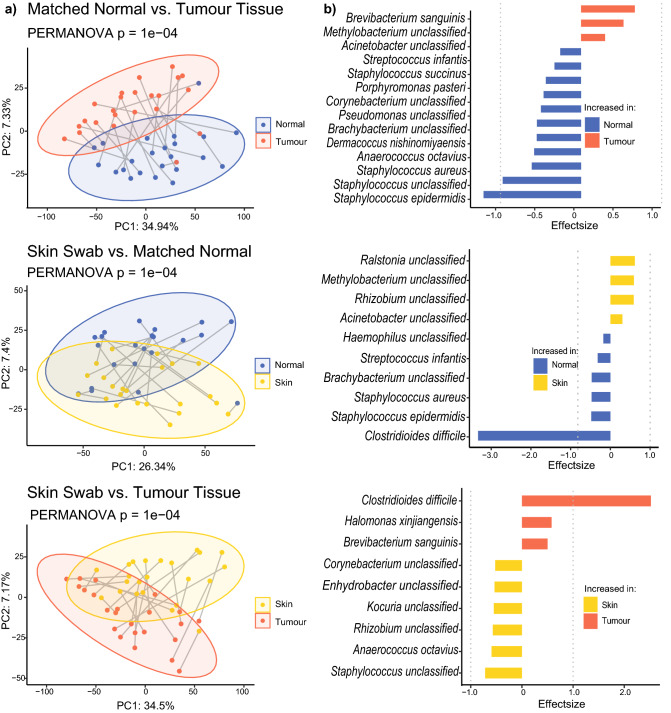
Microbiota composition in tumour and matched normal tissues and skins swabs. **(a)** Principal component analysis, based on Aitchison distances, of all bacterial species that are present in at least 5% of samples. Paired samples are connected by grey lines. (**b)** Differentially abundant taxa with an adjusted p-value of less than 0.05. Effect size is defined as the between-group differences divided by the within-group differences.

To further compare the microbial composition of skin swabs, breast tumour, and matched normal tissue, sequencing reads were collapsed into species level (where possible) and filtered based on presence in at least 5% of the samples. All comparisons showed that all three specimen types had distinct microbial signatures (PERMANOVA *P* = 0.001) (Fig. 3a). Differential abundance analysis with ALDEx2 revealed 11 significantly increased taxa and three decreased taxa in matched normal tissue compared with tumour tissue—most prominently *Staphylococcus epidermidis* and *Brevibacterium sanguinis*, respectively. Six taxa were significantly increased (especially *Clostridoides difficile)* while four taxa were decreased (especially *Ralstonia* spp.) in matched normal breast tissue when compared with skin swabs. Finally, nine taxa were differentially abundant when comparing skin swabs with tumour tissues, with six taxa being increased and three decreased in skin swabs—most importantly *Staphylococcus* spp. and *C. difficile* (Fig. 3b, Supplementary tables 1–3). The presence of some of these bacteria is corroborated by reports from other groups—for example, Clostridia have been shown to be elevated in tumours of patients that respond well to immunotherapy ^7^.

### Machine learning predictions based on bacterial signature are effective in differentiating malignant and non-malignant tissues

**Figure 4:**
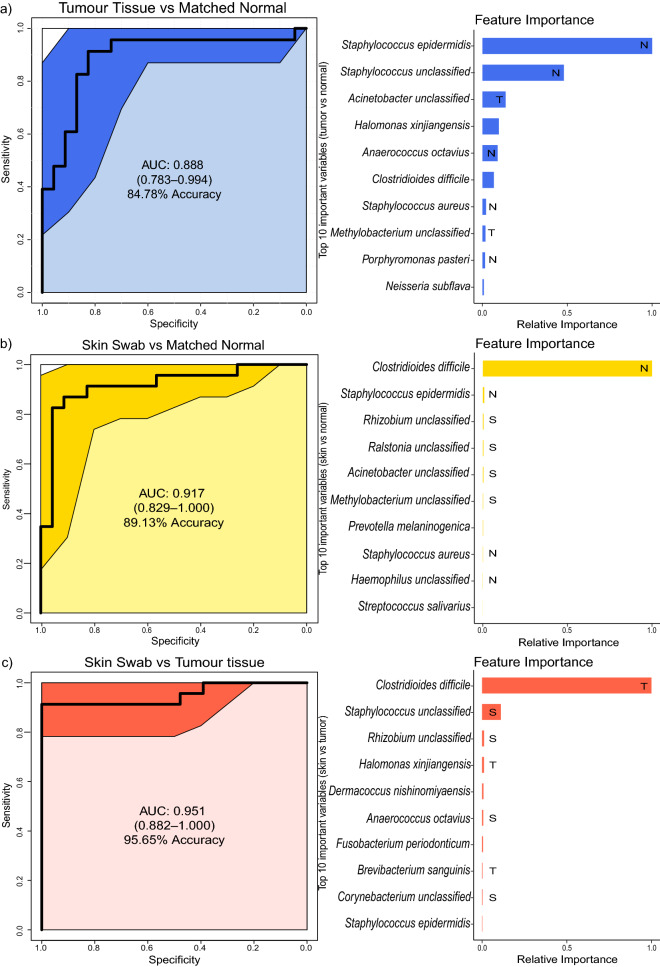
Pairwise machine learning classification of breast cancer patient specimens. Receiver operating characteristic curves (ROC) for the boosted tree models based on species abundance with proportional normalisation on species present in at least 5% of samples. Illustrated are comparisons of: (**a)** tumour and matched normal tissue, (**b)** skin swabs and matched normal tissue, and (**c)** skin swabs and tumour tissue. A model with an area under the curve (AUC) of 0.5 has no discriminatory capacity, whereas an AUC of 1 indicates perfect separation of the response variables. The solid black line tracks the consensus AUC, while the surrounding, shaded area defines the 95% confidence interval. Beside each ROC curve, feature importance plots show the relative importance for the 10 taxa with the highest gain, normalised by the frequency a particular taxon is chosen for a model, for each comparison alongside their highest known classification. The labels within the bars indicate the sample type in which the respective taxa are significantly increased. Bars without labels indicate that the respective taxon is not differentially abundant in any sample type.

The distinctiveness of different patient sample types, in terms of their bacterial profile, was determined using the ‘Extreme Gradient Boosting’ machine learning technique, including bacterial species present in at least 5% of all samples, and proportionally normalised. The binary classifiers were able to distinguish between breast tumour and matched normal tissues (0.888 AUC, 84.78% accuracy), as well as between skin swabs and matched normal tissue (0.917 AUC, 89.13% accuracy) and skin swabs and tumour tissue (0.951 AUC, 95.65% accuracy). While *S. epidermidis* was the most important feature to differentiate between tumour and matched normal tissue, the presence of *C. difficile* allows for extremely accurate discrimination between skin swab samples and both tumour and matched normal tissues (Fig. 4, Supplementary tables 4–6).

## Discussion

There is debate concerning the extent to which microbes are incidental colonisers of tumours, or if they are themselves tumourigenic. Whatever the relationship, the possibility of using microbial profiling to diagnose malignant disease is an attractive concept and its feasibility is considered in this study using a more authentic foundation than what can be provided via TCGA project source material.

The workflow followed in this study was calibrated to minimise the probability of contamination in both a wet laboratory and bioinformatic context using several approaches. First, a progressive contamination control strategy was implemented in line with the RIDE checklist^11^. Second, all patient samples were provided directly by the breast surgeon, from the operating theatre, to laboratory personnel during the patient's surgery. This was a favourable truncation of the traditional procedure, as investigators of the breast microbiome often report relatively high levels of sample manipulation prior to DNA extraction, including excision of the breast specimen, followed by further handling in a pathology laboratory^4,6^. By removing this step, patient tissues were handled by less people over a shorter timeframe and were not exposed to the environmental contamination that might arise in the pathology department. Third, all patient samples were provided by a single surgical team under one consultant breast surgeon, providing a more consistent and reliable foundation for specimen collection.

The results of this can be seen in Fig. 1—approximately 20% of reads had to be discarded as contamination, with sufficient sequencing depth remaining to conduct robust statistical analysis. Comparisons of the overall bacterial community structure at the phylum level prior to and following contamination removal corroborate these findings, suggesting that the bespoke workflow is highly effective at eliminating contamination.

One confounding factor potentially affecting this study included a lack of comparison between tumour tissues and matched tissues taken from non-cancer patients. While the precedent investigation on this topic also did not acquire these data^8^, the diagnostic authenticity of the approach is likely unaffected by this, given that the ability to distinguish between tumour and matched normal tissues within the same patient is probably more powerful than the ability to distinguish between corresponding tissues in cancer and non-cancer patients. Indeed, some microbiome studies have employed matched normal tissues as substitutes for tumour tissues, due to their anticipated similarities in terms of their microbial communities^2,6^. Another potential limitation of this study is that only palpable tumours were biopsied and included in the final selection. This means that very small lesions were excluded from the current cohort, as they were not tangible. Yet, the range of tumour sizes biopsied varied widely, and tumours as small as 0.2 cm were in fact palpable and resected (Table 1). It remains a possibility, though, that tumours smaller than this were filtered out mid-study and are unrepresented in this work. A final, potential complicating factor concerns the different ways in which the breast tumour and matched normal tissues were obtained in this study—via a biopsy needle and diathermia, respectively. While different sampling methodologies could introduce variability and distort data interpretability, it is unlikely in this case that variations in sampling technique introduced significant changes to the tissues in terms of their microbiome composition. This is because both sampling techniques were similar in that they were implemented consecutively during invasive surgery, to sample patient tissues directly with minimal probability of cross-contamination from other tissue types. In fact, even when breast tissues are sampled in a minimally invasive context (i.e., the patient’s skin is contacted) using biopsy needles, and compared with invasive surgical excision biopsies (where the skin is not contacted), the respective microbiomes are not significantly influenced by the sampling technique variation ^5^.

**Table 1:** Biographical, histological, surgical, and medical information for enrolled breast cancer patients.

Demographic and clinical information for breast cancer patients (n = 23)		
Age, median (range)	70 (40–83)	
Sex, n (%)	2 (8.70%)	Male
	21 (91.30%)	Female
Cancer type^a^, n (%)	16 (69.57%)	Ductal invasive
	5 (21.74%)	Lobular invasive
	2 (8.70%)	Invasive solid papillary carcinoma
	1 (4.35%)	Metastatic spindle cell carcinoma
Tumour grade, n (%)	16 (69.57%)	Grade 2
	7 (30.43%)	Grade 3
Surgery type, n (%)	15 (65.22%)	Mastectomy
	8 (34.78%)	Wide local excision
Tumour size in cm, median (range)	3 (0.2–10)	
Largest metastatic focus in cm, median (range)	1.1 (0.1–3)	
Antibiotic use within 1 month prior to surgery^b^, n (%)	2 (8.70%)	
Regular probiotic use^b^, n (%)	11 (47.83%)	
History of breastfeeding^b^, n (%)	6 (26.09%)	
History of adjuvant therapy, n (%)	0 (0%)	
History of neoadjuvant therapy, n (%)	1 (4.35%)	
Tumour necrosis, n (%)	9 (39.13%)	
Calcifications—malignancy-associated, n (%)	8 (34.78%)	
Calcifications—benign tissue-associated, n (%)	11 (47.83%)	
Oestrogen receptor positive, n (%)	21 (91.30%)	
Progesterone receptor positive, n (%)	18 (78.26%)	
HER-2 receptor positive, n (%)	0 (0%)	
Ductal ectasia, n (%)	3 (13.04%)	
Focal lactational change, n (%)	1 (4.35%)	
Lymphovascular invasion, n (%)	12 (52.17%)	
Extranodal extension, n (%)	9 (39.13%)	
Extensive intraductal component, n (%)	1 (4.35%)	
Skin involvement, n (%)	8 (34.78%)	

^a^Some patients had overlapping cancer types (e.g., both lobular carcinoma and ductal invasive carcinoma).

^b^One patient did not provide information.

We have shown that our predictive, machine learning model is accurate when used to determine the malignancy status of human tissue, strongly suggesting that intratumoural bacteria may have the facility to act as cancer biomarkers. The classification accuracy of 84.78% is impressive and compares favourably with established clinical cancer diagnostics that are known to underperform. An example of this is the high false-positive rate observed (between 30 and 87%) when attempting to differentiate ductal carcinoma in situ from benign breast disease^17^. Despite its good performance, it may be premature to pronounce on the true diagnostic utility of our technology, due to the high variability of sequence-based analyses of bacterial communities ^18^. However, with the increasing, widespread availability of bacterial DNA sequence data, from this and other tumour microbiome studies, a sufficiently varied training data set will soon be publicly available to bridge this gap.

Prospective work on this topic should investigate alternative tumour types to establish how broadly a cancer diagnostic approach that incorporates microbial profiling can be applied. It is reported that malignancies beyond breast cancer are associated with a microbiome, and these are being explored for various microbiome-based medical applications. For example, it has been proposed that the pancreatic ductal adenocarcinoma microbiome has the capacity to generate oncogenic signals via tumour immunosuppression, that could be potentially intercepted to disrupt disease progression^19^. Given that diagnostic algorithms for pancreatic adenocarcinoma are poorly defined^20^, the exploration of microbiome data as a diagnostic tool for this cancer is a worthy pursuit. Microbiome research is continually advancing, bringing with it pushes for increased refinement and standardisation in the way data are collected and analysed^21^. As this occurs, the true applicability of these data to health and disease should become clear.

## Materials and methods

### Independent validation of study material

Clinical research was approved by the Clinical Research Ethics Committee (CREC) of University College Cork, Cork, Ireland. All experimental procedures were carried out in accordance with the relevant guidelines and regulations. Breast cancer was confirmed in each patient using a ‘triple assessment’ approach^22^. This protocol is the gold standard for breast cancer diagnosis, incorporating physical examination, imaging (e.g., mammography), and core biopsy. When used individually, each of these modalities is associated with an appreciable degree of unreliability, especially when compared with their use in concert. When combined, triple assessment yields a positive predictive value of 100%, as well as a sensitivity (the extent to which the diagnostic can confirm breast cancer) and specificity (the capacity of the diagnostic to determine the absence of breast cancer) of 94.7% and 100%, respectively. Following a positive diagnosis, it was ensured that tumour biopsies retrieved only tissue from within the patient’s lump by working with palpable masses only (i.e., tumours were not biopsied if they were not palpable). Matched normal tissue was biopsied by removing tissue 3–4 cm from the primary tumour margin.

### Clinical specimen collection and transportation

Approval for this study was received from the Clinical Research Ethics Committee of the Cork Teaching Hospitals (ECM 4 (h) 04/06/13). Informed consent was sought from each patient and/or their legal guardian(s) before their inclusion. 21 female patients and 2 male patients with breast cancer were enrolled in the study. Demographic and clinical information for these patients are detailed in Table 1. Three sample types were retrieved ipsilaterally from each patient: a skin swab, breast tumour tissue, and matched normal breast tissue. Overall, 23 breast tumour samples, 23 matched normal tissue samples, and 23 skin swab samples were obtained from 23 breast cancer patients—i.e., all three specimen types were sampled from every patient. First, the patient's skin was disinfected at their surgical site with ChloraPrep with Tint (CareFusion, USA) and the intact epidermis of the patient’s breast was subsequently swabbed with a sterile gauze pad at the point of surgical incision, prior to surgical incision. The gauze pad was then left exposed to the operating theatre's environment until all samples were collected. Breast tumour tissue was extracted from each patient using a sterile, 14-French biopsy needle (ACHIEVE programmable automatic biopsy system, Merit Medical, USA). This was accomplished by passing the needle through the centre of the tumour during open surgery, prior to resection of the entire tumour by the surgeon. Matched normal tissue was excised from each patient using a sterile diathermy needle, during open surgery also, directly after tumour biopsy. The site at which matched normal tissue was removed was guided by the location of the tumour alone and was consistently resected outside of the marginal zone, between 3 and 4 cm from the edge of the tumour. All tissues and skin swabs were retrieved by a single breast surgeon, and a consistent sampling technique was used for every specimen type. Breast tissues were divided and placed into 30-ml universal containers. Skin swabs were stored and transported in 1 ml reinforced clostridial medium (RCM) (Oxoid, United Kingdom). Samples were transferred from the operating theatre to the laboratory within 20 min of collection. Tubes containing skin swab samples were vortexed, followed by removal of the gauze pad with a sterile forceps. Breast tissues and some volume of RCM from the skin swab samples were flash-frozen and stored in a −80 C freezer for subsequent bacterial DNA extraction. These samples were processed, subsequently passed quality control tests, and proceeded to downstream analyses, as described below.

### DNA extraction, 16S rRNA library preparation, and sequencing

DNA from 23 patient tissues and skin swabs was subjected to 16S rRNA sequencing. DNA was first extracted from flash-frozen patient breast tissue and skin swab samples using the Ultra-Deep Microbiome Prep kit (Molzym, Germany). Skin swabs and tissue samples were processed per ‘Protocol 1’ and ‘Protocol 2’ of the kit manual, respectively. Steps requiring use of a thermomixer were performed using a T-Shaker (EuroClone, Italy) at 1000 rpm. 1 ml Buffer SU was run through the kit as a negative control, per ‘Protocol 1’. In total, 12 sets of DNA extractions were performed, each with a corresponding negative kit control. These negative kit controls were combined into three separate pools, and sequenced, as described below.

Eluted DNA was quantified using a Qubit fluorometer (Invitrogen, USA) using the ‘High Sensitivity’ assay, and PCR-amplified using primers targeting the V3-V4 region of the 16S rRNA gene (forward primer 5'-TCG TCG GCA GCG TCA GAT GTG TAT AAG AGA CAG CCT ACG GGN GGC WGC AG-3' and reverse primer 5'-GTC TCG TGG GCT CGG AGA TGT GTA TAA GAG ACA GGA CTA CHV GGG TAT CTA ATC C-3'). 35-µl reactions were set-up per the following recipe: 17.5 µl NEBNext Ultra II Q5 Master Mix (New England Biolabs, USA), 1.75 µl forward and reverse primers (final concentration: 0.5 µM), and 14 µl template DNA. Two sets of amplicon PCRs were conducted in total, both with corresponding negative controls that were made by replacing 14 µl template DNA in the above recipe with 14 µl microbial DNA-free water (Qiagen, Germany). Both PCR negative controls were sequenced separately, as described below. Reactions were run in a Mastercycler Gradient per the following protocol: 98 C for 30 s, followed by 25 cycles of 98 C for 10 s, 60 C for 30 s, and 72 C for 40 s, followed by a final extension step of 72 C for 5 min. The product was approximately 460 bp.

Reactions were cleaned per the ‘16S Metagenomic Sequencing Library Preparation’ protocol (Illumina, USA), with the exception that samples were dried for 90 s following removal of ethanol, rather than for 10 min. Samples were eluted in 30 µl Buffer EB (Qiagen, Germany). Purified DNA proceeded to index PCR per the Illumina protocol, with the exception that 15 µl template was used, while PCR-grade water was omitted from the recipe. Index PCR products were cleaned per the Illumina protocol, reducing the drying time, as above. DNA quantification was performed using a Qubit fluorometer, as above. Samples were normalised separately by pooling 40 ng DNA per sample. Samples that were too dilute to be normalised to these quantities had their total volume added to the pool. A paired-end, 300-bp run was subsequently completed on an Illumina MiSeq, at GENEWIZ, Inc., USA.

### Bioinformatic data processing

The quality of 2 × 300-bp, paired-end sequence data was initially visualised using FastQC (v0.11.6), and then filtered and trimmed using Trimmomatic (v0.36), to ensure a minimum average quality of 25. The remaining high-quality reads were imported into the R environment (v3.6.2) for processing with the DADA2 package (v1.8.0). DADA2 was used to build an error model used to collapse raw sequences into amplicon sequence variants (ASV), which were then filtered to remove chimeric reads and human-aligning sequences. ASVs were classified to the genus level using the classify.seqs function within the Mothur suite of tools, with species-level resolution provided by SPINGO directed at the most recent SILVA database (v138).

The bioinformatic contamination control tools Decontam (v1.0.0)^16^ and SourceTracker (v1.0)^15^ were used, according to published guidelines^9,14^, to retrospectively assess and remove contamination, based on sequencing data from negative controls.

### Data analysis and visualisation

All statistical analyses were performed in the R environment. Microbial composition was evaluated with “vegan” (v 2.5-7) by performing principal component analysis (PCA) on Aitchison distances, which were calculated with ‘ALDEx2’ (v 2_1.16.0). Differences between sample location were assessed using permutational multivariate analysis of variance (PERMANOVA). ‘ALDEx2’ was used to calculate pairwise differential abundances. To distinguish between tissue sampling sites, *n* leave-one-out gradient-boosted tree models were generated, using “xgboost” (v1.2.0.1). To predict the class of the *n*th sample. Optimal model hyperparameters were determined with bootstrapping of 100 iterations and five-fold cross-validations. The performance of the classification was measured by the area under the ROC curve (AUC), utilising the “pROC” package (v1.16.2). This curve is constructed by plotting the sensitivity, or true positive rate, against the false positive rate, which is calculated as 1-specificity. Feature importance was determined by the ‘gain’ that an included bacterial species added to a model and the frequency with which each species was used for a model.

## Supplementary Information

Supplementary Tables.

## Data Availability

The datasets generated from the current study are available from the corresponding author on reasonable request. All raw sequencing data described in this manuscript is available on ENA/SRA under the accession number PRJEB55383.
