# Author Correction: Biopsy bacterial signature can predict patient tissue malignancy

**DOI:** 10.1038/s41598-022-24161-1

**Published:** 2022-11-15

**Authors:** Glenn Hogan, Julia Eckenberger, Neegam Narayanen, Sidney P. Walker, Marcus J. Claesson, Mark Corrigan, Deirdre O’Hanlon, Mark Tangney

**Affiliations:** 1 Cancer Research@UCC, University College Cork, Cork, Ireland; 2 SynBioCentre, University College Cork, Cork, Ireland; 3 APC Microbiome Ireland, University College Cork, Cork, Ireland; 4 School of Microbiology, University College Cork, Cork, Ireland; 5 General Surgery, Cork University Hospital, Cork, Ireland; 6 General Surgery, South Infirmary Victoria University Hospital, Cork, Ireland

Correction to: *Scientific Reports* https://doi.org/10.1038/s41598-021-98089-3, published online 17 September 2021

The original version of this Article omitted information on data deposition in the Data Availability section, where

“The datasets generated from the current study are available from the corresponding author on reasonable request.”

now reads:

“The datasets generated from the current study are available from the corresponding author on reasonable request. All raw sequencing data described in this manuscript is available on ENA/SRA under the accession number PRJEB55383.”

The original Article has been corrected.

